# Deletion of Circadian Rhythms Gene BMAL1 Impairs the Intestinal Epithelial Barrier and Exacerbates Intestinal Inflammation by Inducing Pyroptosis

**DOI:** 10.1155/mi/9930240

**Published:** 2026-07-22

**Authors:** Xin Zhou, Jie Ji, Wenhua Li, Wenxia Wu, Huanyan Zhang, Pingyu Gao, Wenjiao Ren, Guanzhao Zong, Weiliang Jiang, Rong Wan, Kai Zhao, Yongbin Ma, Zhanjun Lu

**Affiliations:** ^1^ Department of Gastroenterology, Jintan Hospital, Jiangsu University, Changzhou, Jiangsu Province, China, ujs.edu.cn; ^2^ Clinical Laboratory, Jintan Hospital, Jiangsu University, Changzhou, Jiangsu Province, China, ujs.edu.cn; ^3^ Department of Gastroenterology, Jiading Branch of Shanghai General Hospital, Shanghai JiaoTong University School of Medicine, Shanghai, China, shsmu.edu.cn; ^4^ Pathology Center, Jintan Hospital, Jiangsu University, Changzhou, Jiangsu Province, China, ujs.edu.cn; ^5^ Department of Critical Care Medicine, Jintan Hospital, Jiangsu University, Changzhou, Jiangsu Province, China, ujs.edu.cn; ^6^ Department of Gastroenterology, Shanghai General Hospital, Shanghai JiaoTong University School of Medicine, Shanghai, China, shsmu.edu.cn; ^7^ Department of Central Laboratory, Jintan Hospital, Jiangsu University, Changzhou, Jiangsu Province, China, ujs.edu.cn

**Keywords:** BMAL1, circadian rhythms, intestinal epithelial barrier, pyroptosis, ulcerative colitis

## Abstract

The circadian clock plays a crucial role in the pathogenesis of various inflammatory and autoimmune diseases, including ulcerative colitis (UC). Deletion of the core transcription factor BMAL1 exacerbated the severity of colitis. However, the underlying molecular mechanisms of BMAL1 in UC remain unclear. We found that BMAL1 was downregulated in UC tissues and in LPS‐induced MODE‐K cells, whereas CXCL1 was highly expressed. Overexpression of BMAL1 reduced LPS‐induced pyroptosis in MODE‐K cells and restoring the expression of ZO‐1, Claudin‐1, and Occludin, thereby improving intestinal epithelial barrier function. Mechanistically, BMAL1 can negatively regulate the CXCL1 expression by inhibiting the activity of its promoter. Additionally, proteomics analysis identified MEF2A as a downstream protein of BMAL1. The protective effect of BMAL1 on MODE‐K cells was achieved through direct negative regulation of CXCL1 or indirect negative regulation of MEF2A expression. Thus, BMAL1 plays a protective role in maintaining the integrity of the intestinal epithelial barrier and represents a potential therapeutic target for UC treatment.

## 1. Introduction

Ulcerative colitis (UC) is a chronic, idiopathic inflammatory disease that primarily affects the colon and most commonly occurs in adults aged 30–40 years [1–3]. The pathogenesis of UC is multifactorial, with a widely accepted theory suggesting that chronic inflammation in UC is driven by a vicious cycle involving disruption of the intestinal barrier, cellular apoptosis, and a subsequent inflammatory response [4–6]. Therefore, investigating the molecular mechanisms underlying gut barrier disruption is essential to gain understanding of UC pathogenesis.

Intestinal epithelial cells (IECs) play a crucial role in maintaining the integrity and barrier function of the intestinal epithelium, which serves as a physical barrier against the entry of harmful luminal substances, large hydrophilic molecules, and bacterial organisms [7, 8]. Tight junctions (TJs) work synergistically with adherens junctions to connect neighboring IECs and form a selective barrier against paracellular solute permeability [9, 10]. The reconfiguration of TJs by selectively removing or inserting specific proteins can enhance intestinal permeability, potentially resulting in the dysregulation of fluid and electrolyte transport and thereby predisposing to colitis [11]. Previous studies in preclinical models of ulcerative colitis (UC) have demonstrated a significant loss of IECs through abnormal cell death [12, 13]. Furthermore, confocal endoscopy can identify intestinal barrier dysfunction prior to the development of intestinal lesions, enabling prediction of ulcerative colitis recurrence [14]. Additionally, deficiencies in the expression of TJ proteins, such as Claudin‐1 and Claudin‐7, can increase intestinal permeability and promote intestinal injury [15, 16]. This evidence suggests that intestinal epithelial barrier dysfunction is strongly associated with the development of UC.

The circadian clock is a highly conserved endogenous molecular system that regulates circadian rhythms on a 24‐h cycle, ensuring the synchronization of internal physiological processes with changes in the external environment [17]. Circadian rhythms play a vital role in intestinal physiology, including motility, secretion, blood flow, and the integrity of the intestinal barrier [18, 19]. For instance, research has shown that genetic disruption of circadian function or environmental circadian rhythm disturbances (CRDs) in mice increase susceptibility to severe intestinal inflammation and epithelial dysregulation, characterized by excessive necrotic cell death and a reduced number of secretory epithelial cells [20]. Du et al. [21] demonstrated that circadian rhythm dysregulation leads to intestinal barrier disruption and triggers intestinal inflammation. These findings suggest that circadian rhythms are crucial for maintaining the integrity of the intestinal barrier. However, the current literature lacks detailed studies on the specific mechanisms regulating circadian rhythms at the intestinal epithelial barrier.

The aryl hydrocarbon receptor nuclear translocator‐like protein 1 (BMAL1) is a key transcriptional activator of the mammalian biological clock and plays a vital role in the physiological functions of organisms [22]. Previous studies have found that levels of C‐reactive protein (CRP) and fecal calprotectin (Fcal) in patients with UC are negatively correlated with BMAL1 levels in peripheral blood samples, suggesting that BMAL1 may be involved in UC pathogenesis. BMAL1 deficiency was observed in MODE‐K cells and was found to promote colitis [23]. Downregulation of BMAL1 exacerbated dextran sulfate sodium (DSS)‐induced colitis by compromising the intestinal barrier [24]. These findings further demonstrate that BMAL1 deficiency is closely associated with intestinal barrier damage. However, current studies have yet to elucidate the molecular mechanisms by which BMAL1 regulates intestinal barrier integrity.

In this study, we investigated the role and mechanism of BMAL1 in MODE‐K cells. Our findings suggest that BMAL1 plays a crucial role in reducing cell death and enhancing the function of the intestinal epithelial barrier by direct negative regulation of CXCL1 or indirect negative regulation of MEF2A expression. Therefore, targeting BMAL1 could offer a promising therapeutic strategy for UC.

## 2. Materials and Methods

### 2.1. Patients and Sample Collection

All UC patients and controls were recruited at the Jintan Hospital. Of these, a total of 15 patients with a confirmed diagnosis of UC and 15 controls without endoscopic findings of any type of colitis or tumor were included to collect colonic mucosal tissue samples. For the severity of UC, total colonoscopy was used to assess the severity of UC. Biopsies were performed from the rectal segment. The Montreal classification is used to define the extent of UC, E1: ulcerative proctitis (proximal extent of inflammation is located distal to the rectosigmoid junction); E2: left‐sided UC, distal UC (involvement is limited to a portion of the colorectum distal to the spleen); and E3: generalized UC, total colitis (involvement extends to the proximal part of the spleen) [25]. Healthy controls were those who were actively undergoing endoscopy to screen for colon polyps or cancer. Samples from UC or healthy control patients were taken from the most inflamed part of the colon and were immediately stored at −80°C. The study participants agreed to the study protocol. This observational research was also approved by the Ethics Committee of Jintan Hospital (Approval Number: 2023005). Patient information and sample collection times are shown in Supporting Information 1: Table S1.

### 2.2. Quantitative Reverse Transcription PCR (RT‐qPCR)

RNA is isolated from cells or colon tissues using the FastPure Cell/Tissue Total RNA Isolation Kit (BD Biosciences, USA). Subsequently, total RNA was reverse‐transcribed to single‐stranded cDNA using the High‐Capacity cDNA Reverse Transcription Kit (Applied Biosystems, Life Technologies, USA). Fast SYBR Green Master Mix (Applied Biosystems, Life Technologies) was used to amplify the cDNA. The amount of RNA extracted using the StepOnePlusTM was measured using RT‐qPCR. Primer sequences used in this study are displayed in Table 1.

**Table 1:** List of all primers used RT‐qPCR.

Species	Primer	Sequence (5′–3′)
Homo sapiens	GAPDH_F	CCCAAACCGAAGTCATAGC
	GAPDH_R	CGCCCAATACGACCAAAT
	BMAL1‐F	GTGCCACCAATCCATACA
	BMAL1‐R	CCCTCGGTCACATCCTAC
	CLOCK‐F	GTTGAAGAACCCAATGAA
	CLOCK‐R	CAAATGGCAAATACCCTA
	CRY1‐F	TCTCCGATTTGGTTGTTT
	CRY1‐R	GATTATTTGTTGCTGCTGT
	CRY2‐F	ACCACGACGAGACCTACGG
	CRY2‐R	GGAGGGAGTTGGCGTTCA
	CXCL1‐F	CACTGCTGCTCCTGCTCCT
	CXCL1‐R	GGCTATGACTTCGGTTTGG
	PER1‐F	CCAGCCATTCCGCCTAAC
	PER1‐R	GCAGCCCTTTCATCCACAT
	PER2‐F	TTCTCCCATTCGGTTTCG
	PER2‐R	CACCCTGACTTTGTGCCTC
	PGK1‐F	TGAAGATTACCTTGCCTGTT
	PGK1‐R	TCTGCTTAGCCCGAGTGA
Mice	GAPDH_F	TTGACCCTGAAGCTCCCT
	GAPDH_R	CAATCTCCACTTTGCCACT
	BMAL1‐F	GTGCCACCAACCCATACA
	BMAL1‐R	CCCTCGGTCACATCCTAC
	CXCL1‐F	GGCTGGGATTCACCTCAA
	CXCL1‐R	GGCTATGACTTCGGTTTGG
	HDAC8‐F	CAACTGGTCTGGAGGGTG
	HDAC8‐R	ATTTCCGTCGCAATCGTA
	MEF2A‐F	CAGGTGGTGGCAGTCTTG
	MEF2A‐R	TTATCCTTTGGGCATTCA
	STAT5B‐F	GGCTATCCTGGGTTTCGT
	STAT5B‐R	AAAGGCATCAGATTCCAAA
	ZNF281_F	TTGGGCAGAAATCCTTGT
	ZNF281_R	TGCTGGCAGTTGGTAGGT

The fluorescence signal was normalized using an internal reference, and the threshold cycle (*C*t) was set during the exponential period of the PCR. The target gene PCR *C*t values were normalized by subtracting the GAPDH *C*t value to obtain the Δ*C*t value. The relative expression level of each target between groups was then calculated using the following formula: relative gene expression = 2^−(△*C*t sample−△*C*t control)^.

### 2.3. Western Blotting Analysis (WB)

The whole protein was extracted from cells or colon tissues using RIPA lysis buffer. (Beyotime, China). The proteins are then transferred to a polyvinylidene difluoride (PVDF) membrane by electrophoresis using a 7.5% polyacrylamide gel. The membrane was blocked in 5% goat serum for 1 h, then the primary antibody was added and incubated overnight at 4°C. The next day, the membrane was incubated with a secondary antibody for 1 h. Afterwards, the membrane was incubated with the secondary antibody for 1 h. Develop using the ECL chemiluminescence kit. Table 2 displays the antibodies utilized in this investigation.

**Table 2:** Antibody information.

Experimental items	Gene name	Catalog number	Manufacturer	Dilution ratios
WB	CXCL1	ab307589	Abcam	1:1000
	CLOCK	ab3517	Abcam	1:1000
	BMAL1	ab230822	Abcam	1:1000
	CRY1	ab54649	Abcam	1:1000
	CRY2	ab155255	Abcam	1:1000
	PER1	ab136451	Abcam	1:1000
	PER2	ab179813	Abcam	1:1000
	ZO‐1	ab190085	Abcam	1:1000
	Claudin‐1	ab211737	Abcam	1:1000
	Occludin	ab216327	Abcam	1:1000
	NLRP3	ab270449	Abcam	1:1000
	GSDMD	AF4012	Affinity	1:1000
	c‐caspase‐1	AF4005	Affinity	1:2000
	IL‐1β	ab283822	Abcam	1:1000
	ASC	ab307560	Abcam	1:1000
	MEF2A	ab76063	Abcam	1:1000
	P65	ab32536	Abcam	1:1000
	p‐P65	ab76302	Abcam	1:1000
	GAPDH	AF7021	Affinity	1:3000
	Goat anti‐rabbit	S0001	Affinity	1:3000
IHC	CXCL1	ab307589	Abcam	1:300
	BMAL1	ab230822	Abcam	1:300
	Goat anti‐rabbit IgG (H + L) HRP	S0001	Affinity	1:200
IF	MEF2A	ab76063	Abcam	1:1000
	Goat anti‐rabbit IgG (H + L) FITC‐conjugated	S0008	Affinity	1:200

### 2.4. Mouse Models

Ten C57BL/6 mice (male, 6–8 weeks old) were used in this study and housed in the specific pathogen free (SPF) room at the Jintan Hospital Animal Center. After acclimatizing for more than 1 week, the 10 mice were randomly divided into two groups: the control group and the DSS group, with 5 mice in each group. Except for the control group, mice were given 3% (w/v) dextran sodium sulfate (DSS, 36,000–50,000 mol.wt; MP Biomedicals, USA) in drinking water for 7 consecutive days to induce colitis. DSS‐stimulated mice were randomly divided into NC and BMAL1‐OE groups. For the BMAL1‐OE group, we used a lentiviral (50 μL; 1 × 10^8^ TU) construct driving BMAL1 overexpression specifically in IECs under the control of the Villin promoter, which was administered by intracolonic injection in 3 fractions (days 0, 2, and 4 during DSS treatment). It should be noted that the epithelial specificity of this approach is inferred, as direct validation (e.g., by co‐immunofluorescence) was not performed. On the 21 days, all mice were euthanized by asphyxiation with excess carbon dioxide, and colon tissues were collected. All procedures of animal experiments were approved by the Shanghai General Hospital Clinical Center Laboratory Animal Welfare & Ethics Committee (Approval Number: 2025AWS096). The animal experiments in this study followed the ARRIVE reporting guidelines.

### 2.5. Evaluation of Disease Activity Index (DAI) Score to Assess Disease Severity

To assess the severity of colitis, body weight, fecal character, and blood in the stool were measured according to a previously published grading system [26, 27]. Briefly, weight loss was scored as follows: 0, no weight loss; 1, 1% to 5%; 2, 5% to 10%; 3, 10% to 20%; and 4, >20%. Diarrhea was scored as follows: 0, normal; 2, loose stools; 4, watery diarrhea. Blood in the stool was scored as follows: 0 points, normal; 2 points, slight bleeding; 4 points, heavy bleeding.

### 2.6. Tissue Sections and Immunohistochemical (IHC) Staining

The mouse colon tissue samples were immersed in 4% paraformaldehyde overnight for fixation, then embedded in paraffin and cut into sections that were 4‐μm thick. The sections were stained with hematoxylin and eosin (H&E; Solarbio, China) according to the manufacturer’s protocol. For IHC staining, sections were incubated overnight at 4°C with primary antibody against BMAL1 (1:200) and CXCL1 (1:200). After washing with PBS, sections were incubated with HRP antibody at 37°C for 30 min. Specific antibody information is displayed in Table 2. The sections were then stained with a DAB kit for 10 min and observed under a light microscope (Zeiss, Germany).

### 2.7. Cell Culture and Treatment

The MODE‐K mouse IEC line was obtained from Shanghai GuanDao Biological Engineering (Shanghai, China). Subsequently, cells were grown in Eagle’s Minimum Essential Medium supplemented with 10% FBS (Invitrogen, Carlsbad, USA), 2 mM L‐glutamine, 100 IU penicillin, and 100 mg/mL streptomycin. Cells were incubated in a humidified incubator (containing 5% CO_2_) at 37°C. MODE‐K cells were incubated with 1 μg/mL LPS (Sigma–Aldrich, USA) for 4 h to mimic UC.

### 2.8. Lentiviral Infection

Lentiviral vectors (shBMAL1, shCXCL1, and shMEF2A) interfering with the expression of BMAL1, CXCL1, and MEF2A as well as the negative control shCtrl were synthesized by GeneChem (China). In addition, BMAL1 overexpression (BMAL1) and control (NC) sequences were also designed by GeneChem. MODE‐K cells were transfected with lentiviral vectors in the presence of 5 g/mL polyglutamine (Sigma–Aldrich) for 7 days before treatment with LPS and then screened with puromycin (Sigma– Aldrich) for 7 days, and puromycin‐resistant cells were isolated for further study. Fluorescence plots of cell transfection efficiency after stable lentiviral infection are shown in Supporting Information 2: Figure S1.

### 2.9. Enzyme‐Linked Immunosorbent Assay (ELISA)

According to the manufacturer’s instructions, the commercial Mouse lL‐1β ELISA Kit (SEKM‐0002, Solarbio, China), Mouse TNF alpha ELISA Kit (abs520010, absin, USA), and Mouse Interleukin‐6 ELISA Kit (SEKM‐0007, Solarbio) were used to determine the concentrations of lL‐1β, TNF‐α, and IL‐6 in the culture supernatant, respectively. Finally, the OD values of the samples at 450 nm were measured using a microplate reader (Bio‐Rad, Hercules, USA).

### 2.10. Flow Cytometry

For the determination of apoptosis, cells after transfection or treatment were collected, washed twice with ice‐cold PBS, and resuspended in 1× Annexin V binding buffer. According to the instructions of the Annexin V‐FITC Apoptosis Detection Kit (KeyGen Biotech, USA), cells were stained with 5 μL of Annexin V‐FITC and 5 μL of propidium iodide (PI) and then analyzed by flow cytometry (BD Biosciences). The apoptosis rate was calculated as the sum of early apoptotic (Annexin V‐positive/PI‐negative) and late apoptotic (Annexin V‐positive/PI‐positive) cells.

For the determination of pyroptosis, the FAM‐FLICA caspase‐1 Detection Kit (ImmunoChemistry, Bloomington, MN, USA) was used. Cells were subjected to double staining with PI and FAM‐YVAD‐FMK, and changes in pyroptosis were assessed by flow cytometry. The pyroptosis rate was defined as the percentage of cells positive for both caspase‐1 and PI.

### 2.11. Dual Luciferase Assay

The target gene CXCL1‐promotor was synthesized by Hunan Fenghui Biotechnology Co. (China) and cloned into a PGL3‐basic recombinant vector. 2 × 10^4^ cells/well of MODE‐K cells were cultured in 24‐well plates. Afterward, cells were co‐transfected with either shBMAL1 or shNC and luciferase reporter plasmid using Lipofectamine 2000 (Invitrogen Life Technologies, Carlsbad, CA, USA). Fluorescence activity was detected 48 h after transfection using the Dual‐Luciferase Reporter Promega E1910 Assay System (Promega, USA). Relative fluorescence density was calculated.

### 2.12. Chromatin Immunoprecipitation (ChIP) Assay

The ChIP assay was performed using a commercial kit (ab500, Abcam, Cambridge, UK) according to the manufacturer’s protocol. Cells were cultured in 10 cm dishes until reaching 90% confluence. After cell lysis, the samples were incubated overnight with BMAL1 antibody or IgG at 4°C with rotation for immunoprecipitation. Following DNA purification, quantitative PCR was conducted to assess the enrichment of the CXCL1 promoter region.

### 2.13. iTRAQ Labeling and LC‐MS/MS Analysis

Twenty‐four hours after inoculation, MODE‐K cells were treated with shBMAL1 or shCtrl. Subsequently, cells were harvested with trypsin/EDTA, and total protein was extracted using a lysis buffer containing 1% SDS (Bioman, USA), 50 mM Tris–HCl (pH 6.8), 10% glycerol, and 1× protease inhibitor (Bioman). Proteins were processed overnight by reduction, alkylation, and gel‐assisted tryptic digestion to produce peptides, which were then extracted from the gel as previously described [28]. Equal amounts of control and TIIA‐treated samples were labeled with different iTRAQ reagents (AB SCIEX; control samples were labeled with 114 or 115; TIIA‐treated samples were labeled with 116 or 117) and incubated for 1 h at room temperature. The peptides were mixed and dried using a centrifugal evaporator (CVE‐2000; EYELA, USA). iTRAQ‐labeled samples were desalted and analyzed using an LC‐ESI‐Q‐TOF mass spectrometer (Waters SYNAPT G2 HDMS; Waters Corp.). Samples were injected onto a 180 mm × 2 cm capillary trap column and separated using a nanoACQUITY Ultra Performance LC system (Waters Corp., USA) through a 75 mm × 25 cm nanoACQUITY UPLC 1.7 mm vinyl‐bridged hybrid C18 column. The mass spectrometer (MS) was operated in the electrospray ionization sensitivity mode and calibrated using a synthetic human [Glu1]‐fibrinopeptide B solution (1 pmol/ml; Sigma–Aldrich, USA) delivered through a NanoLockSpray source, which was used for precise mass measurements. Data were acquired in data‐directed analysis (DDA) mode, which consisted of one full MS scan (m/z 350–1700, 1 s) and three consecutive MS/MS scans (m/z 100–1990. 1.5 s per scan) for the three strongest ions present in the full‐scan mass spectrum [29].

### 2.14. Protein Identification and Relative Quantification

Raw data were analyzed using LC‐MS/MS iTRAQ technology with ProteinPilot Software 4.5 (AB Sciex) [29, 30]. Protein identification utilized the human SwissProt_2014_08. fasta sequence database. Searches were performed using a standard set of parameters, including Cys alkylation of methyl methanethiosulfonate (MMTS), biomodification ID focus, trypsin digestion, *Homo sapiens*, search effort, and outright IDs. More than two unique peptides were required for protein identification. Protein identification and quantitative analysis were performed using confidence thresholds higher than 95% and local FDRs less than 1%. Relative quantitation required a *p*‐value <0.05.

Using the limma package in the R language, we identified proteins that exhibited differential expression compared to the shCtrl group. The screening regulation is as follows: *p*  < 0.05 and |long2 (fold change) | >1. The volcano plot was drawn by ggplot2 from the R language. Kyoto Encyclopedia of Genes and Genomes (KEGG) and Gene Ontology (GO) enrichment was implemented by ClusterProfiler (v3.8.1).

### 2.15. Cell Counting Kit 8 (CCK‐8)

Cell viability was determined by Cell Counting Kit 8 (CCK‐8). In brief, cells from MODE‐K cells from different groups were seeded into 96‐well plates at a density of 1 × 10^4^ cells/well and incubated for 24 h. A quantity of 10 μL of CCK8 solution (Beyotime) was added to the medium at 1, 2, 3, 4, and 5 days. Sample absorbance was measured at 450 nm after 4 h of incubation at 37°C using a microplate reader (Bio‐Rad, USA).

### 2.16. Immunofluorescence (IF)

Cells from different groups were inoculated in 24‐well plates covered with glass coverslips and cultured at 2 × 10^4^ cells per well. After treatment, 1 mL of 4% paraformaldehyde was added and washed with PBS. Then incubated with primary antibodies against MEF2A at 4°C overnight. After washing with PBS, they were incubated with anti‐mouse IgG (H + L) FITC‐conjugated for 1 h. After inhibition with DAPI (Sigma), the stained coverslips were fixed and observed under a fluorescence microscope (Zeiss). Specific antibody information is displayed in Table 2.

### 2.17. Statistical Analysis

The GraphPad Prism software 8.0 (USA) was used to analyze all of the data. All experiments were performed with three biological replicates, and the results were presented as mean ± standard deviation (SD). Student’s *t*‐test was used to assess the significance of comparisons between two groups, and one‐way ANOVA combined with Dunnett’s multiple comparison test was used to determine whether there were statistically significant differences in multiple comparisons. A *p* value of less than 0.05 indicated statistical significance.

## 3. Results

### 3.1. Circadian Rhythm Gene Expression was Disrupted in Patients With UC

**Figure 1:**
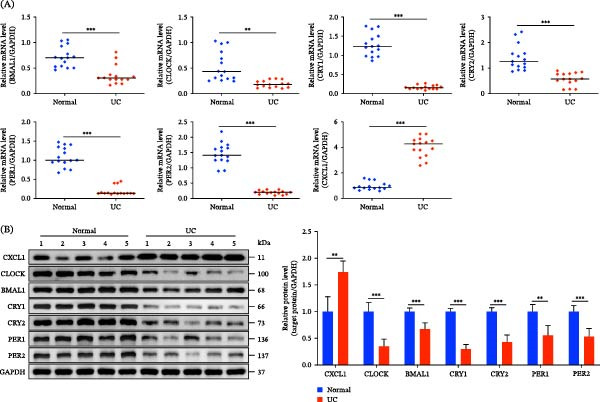
Circadian rhythm gene expression was disrupted in patients with UC. (A) The mRNA expression of CLOCK, BMAL1, CRY1, CRY2, PER1, PER2, and CXCL1 in patients with UC was determined by qRT‐PCR (*n* = 15). (B) The protein expression of CLOCK, BMAL1, CRY1, CRY2, PER1, PER2, and CXCL1 was determined by WB in patients with UC (*n* = 5). Data are presented as mean ± SD ( ^∗∗^ *p* < 0.01,  ^∗∗∗^ *p* < 0.001).

Circadian rhythm disruption has been shown to impair colonic tissue homeostasis and exacerbate chronic inflammation in the colon [20, 31]. Based on the existing literature, the expression of clock genes (CLOCK, BMAL1, CRY1, CRY2, PER1, and PER2) expression has been analyzed in peripheral blood samples from patients with enterocolitis [23]. Accordingly, we selected CLOCK, BMAL1, CRY1, CRY2, PER1, and PER2 to study their expression in colonic tissues of patients with UC. Our findings revealed that the mRNA and protein expression levels of these core circadian clock genes CLOCK, BMAL1, CRY1, CRY2, PER1, and PER2 were significantly downregulated in the colon tissues of UC patients (Figure 1A,B). Chemokine CXCL1, a key gene closely associated with immune cells, is frequently aberrantly expressed in UC [32, 33]. The abnormal expression of CXCL1 plays a significant role in the pathogenesis and progression of ulcerative colitis. Moreover, the circadian rhythm gene can regulate CXCL1 expression [34]. Consequently, in this study, we also analyzed CXCL1 expression levels in the colonic tissues from patients. We found that CXCL1 was significantly upregulated in the UC group compared to the normal group (Figure 1A,B). Furthermore, a literature review indicated that BMAL1 deletion is closely associated with the pathogenesis of colitis [24] and may lead to upregulation of CXCL1 expression [35]. Therefore, we selected the BMAL1 and CXCL1 genes for further investigation. Taken together, these results suggest that dysregulation of circadian rhythm genes in the colonic tissues of UC patients is significant.

### 3.2. Expression Analysis of BMAL1 and CXCL1 in Animal Models of UC

**Figure 2:**
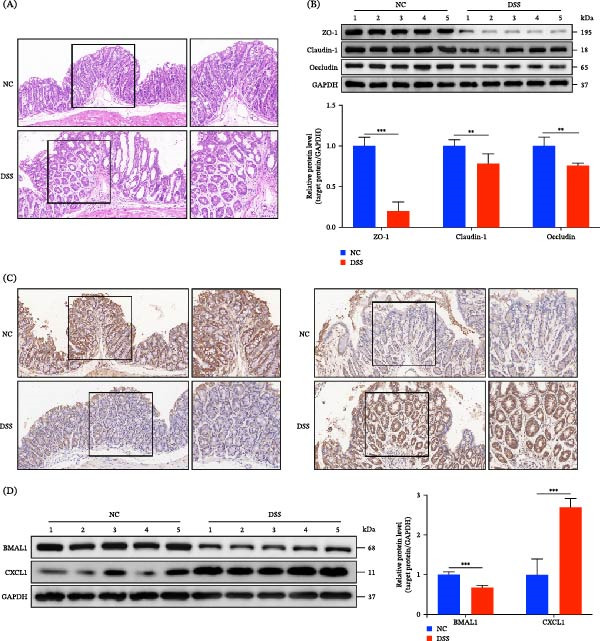
BMAL1 and CXCL1 gene expression in DSS‐induced mice. C57BL/6 mice (male, 6–8 weeks old) were equally divided into 2 groups, mice treated without DSS treatment(NC group, *n* = 5, and mice injected with DSS(DSS group, *n* = 5), with 5 mouse in each group. (A) Representative images of H&E staining of colon tissues (*n* = 5). (B) The protein expression of ZO‐1, Claudin‐1, and Occludin in colon tissues was determined by WB (*n* = 5). (C) Immunohistochemical detection of BMAL1 and CXCL1 in different groups (*n* = 5). (D) The protein expression of BMAL1 and CXCL1 in colon tissues was determined by WB (*n* = 5). Data are presented as mean ± SD ( ^∗∗^ *p* < 0.01,  ^∗∗∗^ *p* < 0.001).

To verify the expression of BMAL1 and CXCL1 in the mouse model, we induced experimental colitis in mice using DSS. The results demonstrated a significant disruption of the epithelial crypt structure, accompanied by an increased infiltration of immune cells into the colon tissues in the DSS‐treated group compared to the untreated control group (NC) (Figure 2A). Additionally, as an indicator of the disruption of intestinal TJs, the protein expression levels of ZO‐1, Claudin‐1, and Occludin in the colon tissues of the DSS group were significantly decreased (Figure 2B), indicating the disruption of intestinal TJs. These findings confirmed the successful establishment of DSS‐induced colitis in mice. IHC and WB were employed to assess the expression of BMAL1 and CXCL1 in the colon tissues of DSS‐induced mice. The results revealed a significant decrease in BMAL1 expression and a notable increase in CXCL1 expression in the DSS group compared to the NC group (Figures 2C,D). Importantly, these results were consistent with those obtained from the clinical samples. In summary, these studies demonstrate that DSS induces intestinal epithelial barrier damage in mice, increases CXCL1 expression, and reduces BMAL1 expression.

### 3.3. Expression Analysis of BMAL1 and CXCL1 in LPS Induced‐Intestinal Epithelial Cells

**Figure 3:**
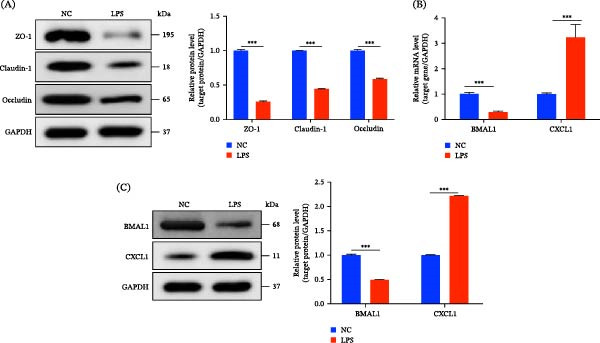
BMAL1 and CXCL1 gene expression in LPS‐induced IECs. Mouse intestinal epithelial cells MODE‐K were divided into 2 groups, the NC group (no LPS treatment) and the LPS group (1 μg/mL LPS for 4 h). (A) The protein expression of ZO‐1, Claudin‐1, and Occludin was determined by WB (*n* = 3). (B) The mRNA expression of BMAL1 and CXCL1 was determined by qRT‐PCR (*n* = 3). (C) The protein expression of BMAL1 and CXCL1 was determined by WB (*n* = 3). Data are presented as mean ± SD ( ^∗∗∗^ *p* < 0.001).

In addition, lipopolysaccharide (LPS) was used to stimulate mouse intestinal epithelial MODE‐K cells to create a model that mimics UC for validating the expression of the circadian rhythm genes BMAL1 and CXCL1. The expression levels of ZO‐1, Claudin‐1, and Occludin were observed to be downregulated upon LPS stimulation (Figure 3A), indicating damage to the MODE‐K cells and confirming the successful establishment of the UC cell model. LPS stimulation was found to downregulate both mRNA and protein expression levels of BMAL1, while concurrently upregulating CXCL1 expression (Figure 3B,C). These findings are consistent with clinical and animal studies. Collectively, the results indicate that the expression of the circadian rhythm gene BMAL1 is reduced in LPS‐induced IECs, whereas the CXCL1 expression is significantly elevated.

### 3.4. BMAL1 Ameliorated LPS‐Induced Intestinal Epithelial Cell Injury and Pyroptosis

To investigate the potential protective role of BMAL1 in MODE‐K cells, we transfected LPS‐induced MODE‐K cells with plasmid vectors to create BMAL1‐overexpressing cells. The results from RT‐qPCR and WB indicated that the transfection of BMAL1 vectors led to an increase in BMAL1 expression and negatively regulated the expression of CXCL1 in the MODE‐K cells (Figure 4A,B). BMAL1 significantly decreased the levels of inflammatory factors IL‐1β, TNF‐α, and IL‐6 in the supernatant of these cells (Figure 4C). In addition, BMAL1 overexpression also reduced LPS‐induced apoptosis in MODE‐K cells (Figure 4D). Meanwhile, BMAL1 overexpression decreased the pyroptosis rate in LPS‐induced MODE‐K cells (Figure 4E). Since LPS can induce pyroptosis in MODE‐K cells, which impairs intestinal barrier function, we also examined the expression of pyroptosis‐related factors. The results demonstrated that BMAL1 could decrease the protein expression of the pyroptosis factors NLRP3, Gasdermin D, and cleaved caspase 1, which were elevated by LPS (Figure 4F). Furthermore, BMAL1 restored the expression of barrier‐associated proteins ZO‐1, Claudin‐1, and Occludin in LPS‐induced MODE‐K cells (Figure 4F). The above findings provide conclusive evidence that BMAL1 exerts a protective effect on LPS‐induced MODE‐K cells, thereby mitigating MODE‐K cell damage and pyroptosis.

Figure 4BMAL1 ameliorated LPS‐induced intestinal epithelial cell injury and pyroptosis. Mouse intestinal epithelial cells MODE‐K were divided into 2 groups, the LPS group (1 μg/mL LPS for 4 h) and the LPS + BMAL1 group (cells were infected with lentivirus overexpressing BMAL1 and treated with LPS). (A) The mRNA expression of Z BMAL1 and CXCL1 was determined by qRT‐PCR (*n* = 3). (B) The protein expression of BMAL1 and CXCL1 was determined by WB (*n* = 3). (C) The levels of IL‐1β, TNF‐α, and IL‐6 were determined by ELISA (*n* = 3). (D) Pyroptosis rate was assessed by flow cytometry (*n* = 3). (E) Apoptosis was assessed by flow cytometry (*n* = 3). (F) The protein expression of NLRP3, Gasdermin D, cleaved caspase‐1, ZO‐1, Claudin‐1, and Occludin was determined by WB (*n* = 3). Data are presented as mean ± SD ( ^∗∗∗^ *p* < 0.001).

**Figure:**
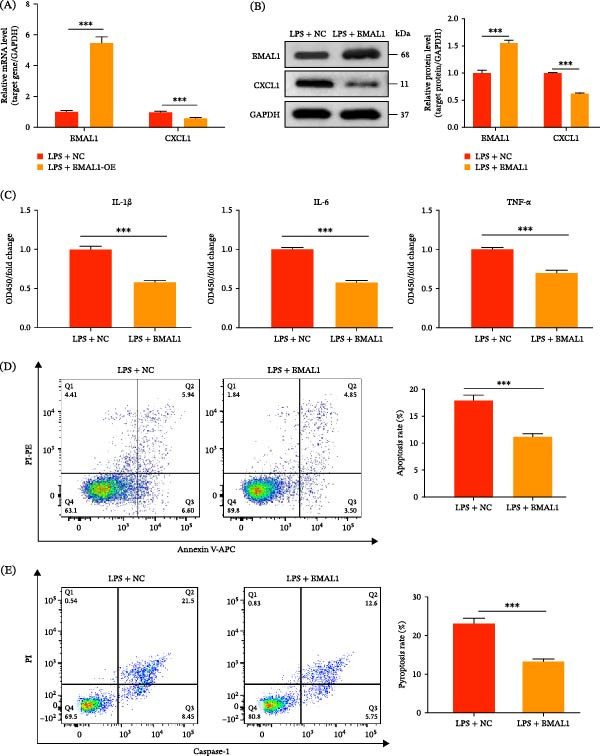

**Figure:**
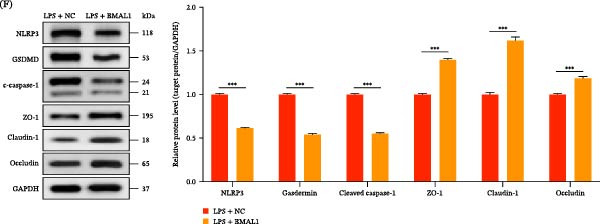

### 3.5. BMAL1 Attenuated LPS‐Induced Intestinal Barrier Disruption by Regulating the Expression of CXCL1

Based on previous results, we found that BMAL1 could negatively regulate the mRNA and protein expression of CXCL1 (Figure 4A,B). However, it remains unclear whether the intestinal protective effect of BMAL1 was dependent on CXCL1 expression. Therefore, in this study, short hairpin RNAs (shRNAs) targeting BMAL1 or CXCL1 were transfected into MODE‐K cells to investigate the roles of BMAL1 and CXCL1. We observed that shBMAL1 induced a significant downregulation of BMAL expression and an upregulation of CXCL1 expression. Furthermore, the upregulation of CXCL1 induced by shBMAL was reversed by shCXCL1 (Figure 5A,B). Flow cytometry results showed that knockdown of BMAL1 induced increased apoptosis in MODE‐K cells, while knockdown of CXCL1 partially reversed this change (Figure 5C). In addition, the knockdown of BMAL1 resulted in increased levels of the inflammatory factors IL‐1β, TNF‐α, and IL‐6 compared to the shCtrl group (Figure 5C). Conversely, the knockdown of CXCL1 exhibited an inhibitory effect on IL‐1β, TNF‐α, and IL‐6 (Figure 5C). Flow cytometry results showed that knockdown of BMAL1 induced an increased apoptosis rate in MODE‐K cells, while knockdown of CXCL1 partially reversed this change (Figure 5D). Notably, knockdown of BMAL1 also increased the pyroptosis rate in MODE‐K cells, and this effect was partially reversed by knockdown of CXCL1 (Figure 5E). Subsequently, we analyzed the factors associated with pyroptosis. Our findings indicated that the knockdown of BMAL1 led to an increase in the expression levels of NLRP3, Gasdermin D, and cleaved caspase‐1, a change that was reversed by shCXCL1 (Figure 5F). Furthermore, the expression of barrier‐associated proteins ZO‐1, Claudin‐1, and Occludin was diminished by shBMAL1; however, shCXCL1 was able to restore the expression of these proteins (Figure 5F). To gain insight into the molecular mechanism by which BMAL1 negatively regulated CXCL1 expression, we conducted a dual‐luciferase reporter assay. The results revealed that in MODE‐K cells, the knockdown of BMAL1 significantly increased the activity of the CXCL1 promoter region (Figure 5G). This suggested that BMAL1 might negatively regulate CXCL1 by blocking the promoter activity of CXCL1. Furthermore, the ChIP assay revealed a significant enrichment of the CXCL1 promoter fragment in the anti‐BMAL1 antibody group compared to the IgG control group (Figure 5H). Knocking down BMAL1 reduced its enrichment at the CXCL1 promoter (Figure 5H). This result indicates that BMAL1 directly binds to the CXCL1 promoter in MODE‐K cells. In conclusion, the above experimental results demonstrated that BMAL1 attenuated LPS‐induced intestinal barrier disruption and pyroptosis by direct regulating CXCL1 expression.

Figure 5BMAL1 attenuated LPS‐induced intestinal barrier disruption by regulating the expression of CXCL1. The LPS‐induced MODE‐K cells were divided into 4 groups, the shCtrl group (cells infected with negative control lentivirus), the shBMAL1 group (Cells infected with shBMAL1 lentivirus), the shCXCL1 group (cells infected with shCXCL1 lentivirus), and the shBMAL1+ shCXCL1 group (cells infected with shBMAL1 and shCXCL1 lentivirus). (A) The mRNA expression of BMAL1 and CXCL1 was determined by RT‐qPCR (*n* = 3). (B) The protein expression of BMAL1 and CXCL1 was determined by WB (*n* = 3). (C) The levels of inflammatory factors IL‐1β, TNF‐α, and IL‐6 were determined by ELISA (*n* = 3). (D) Apoptosis was assessed by flow cytometry (*n* = 3). (E) Pyroptosis rate was assessed by flow cytometry (*n* = 3). (F) The protein expression of NLRP3, Gasdermin D, cleaved caspase‐1, ZO‐1, Claudin‐1, and Occludin was determined by WB (*n* = 3). (G) The relative fluorescence intensity on the CXCL1 promoter was analyzed by a dual‐luciferase reporter assay (*n* = 3). (H) The binding of BMAL1 to the CXCL1 promoter was analyzed by ChIP‐qPCR (*n* = 3). Data are presented as mean ± SD ( ^∗^ *p* < 0.05,  ^∗∗^ *p* < 0.01,  ^∗∗∗^ *p* < 0.001).

**Figure:**
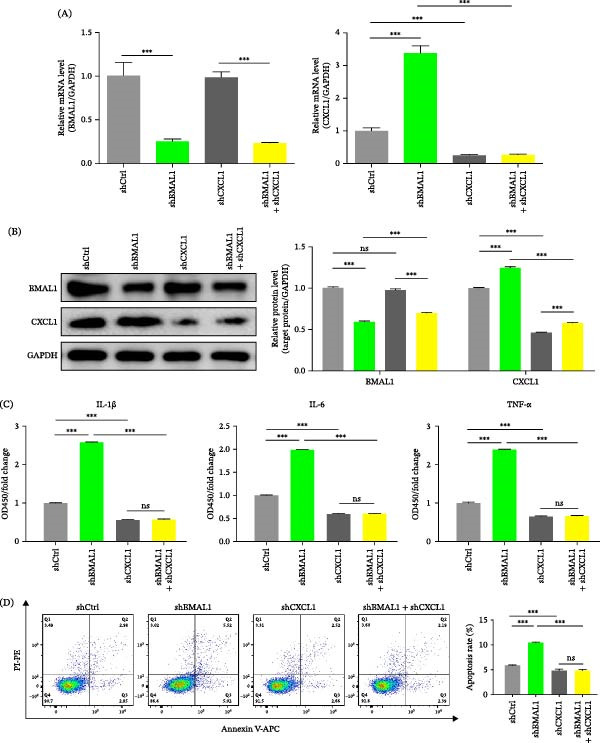

**Figure:**
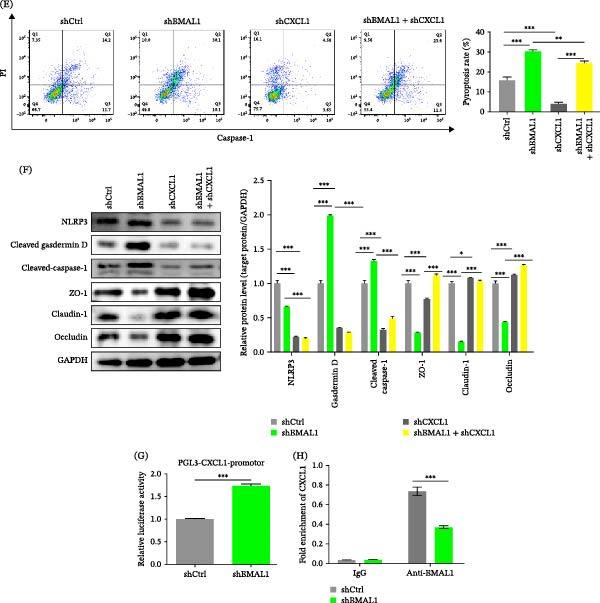

### 3.6. BMAL1 Ameliorated LPS‐Induced Intestinal Epithelial Cell Barrier Damage and Pyroptosis by Regulating the Expression of MEF2A

To elucidate the key proteins regulated by BMAL1, we investigated the differentially expressed proteins in MODE‐K cells following BMAL1 knockdown, based on proteome sequencing analysis. A total of 485 upregulated and 557 downregulated differentially expressed proteins, believed to be regulated by BMAL1, were identified in comparison to the nonknockdown group (Supporting Information 3: Figure S2A and Supporting Information 4: Table S2). Subsequently, these differentially expressed proteins underwent enrichment analysis. The results indicated that these proteins were closely associated with biological processes such as “mitochondrial translation,” “oxidative phosphorylation” and “mitochondrial ATP synthesis coupled electron transport,” and pathways including “Sulfur relay system,” “Proteasome,” “RNA degradation,” and so on (Supporting Information 3: Figure S2B,C). We selected Top4 differentially expressed proteins for RT‐qPCR verification based on protein sequencing results. Compared with shCtrl, the knockdown of BMAL1 significantly increased the expression of HDAC8, STAT5B, and MEF2A, while decreasing the expression of ZNF281 (Supporting Information 3: Figure S2D). Among the factors examined, the trend of MEF2A changes was particularly significant. Additionally, MEF2A is closely associated with the regulation of circadian rhythms [36] and can also trigger inflammatory responses [37]. Consequently, MEF2A was chosen for the cell function study in this research.

First, we transfected MODE‐K cells treated with LPS using shBMAL1 or shMEF2A to establish knockdown models. The results from RT‐qPCR and WB analyses indicated that BMAL1 negatively regulated the expression of MEF2A and CXCL1, while shMEF2A reduced the levels of both MEF2A and CXCL1 (Figure 6A,B). IF results further demonstrated that BMAL1 knockdown decreased the fluorescence intensity of MEF2A, whereas MEF2A knockdown reversed this effect (Figure 6C). Additionally, we observed that MEF2A was localized in the nucleus (Figure 6C). Subsequently, we employed the CCK‐8 assay to evaluate the proliferation of MODE‐K cells. Our findings revealed that BMAL1 knockdown inhibited cell viability, whereas the MEF2A downregulation exerted an opposite effect (Figure 6D). Furthermore, the expression of barrier‐associated proteins ZO‐1, Claudin‐1, and Occludin, which were reduced by shBMAL1, was restored by shMEF2A (Figure 6E). Since MEF2A is a factor regulated by inflammation, we also analyzed the expression of proteins associated with the inflammatory pathway, such as the P65 pathway. The results indicated that shBMAL1 increased P65 phosphorylation levels, while MEF2A knockdown reversed this phosphorylation (Figure 6E), suggesting that MEF2A knockdown may possess anti‐inflammatory potential. Moreover, ELISA results indicated that shBMAL1 increased inflammatory factor levels, which were reversed by MEF2A knockdown (Figure 6F). Finally, we also analyzed the expression of pyroptosis‐related proteins. As expected, knockdown decreased the expression of NLRP3, IL‐1β, and ASC, which were significantly elevated by shBMAL1 (Figure 6G). These findings suggest that BMAL1 exerts a protective effect against LPS‐induced injury and barrier disruption in MODE‐K cells by indirect negatively regulating the expression of the downstream protein MEF2A.

Figure 6BMAL1 ameliorated LPS‐induced intestinal epithelial cell barrier damage and pyroptosis by regulating the expression of MEF2A. The LPS‐induced MODE‐K cells were divided into 4 groups, the shCtrl group (cells infected with negative control lentivirus), the shBMAL1 group (cells infected with shBMAL1 lentivirus), the shMEF2A group (cells infected with shMEF2A lentivirus), and the shBMAL1+ shMEF2A group (cells infected with shBMAL1 and shMEF2A lentivirus). (A) The mRNA expression of BMAL1, CXCL1, and MEF2A was determined by RT‐qPCR (*n* = 3). (B) The protein expression of BMAL1, CXCL1, and MEF2A was determined by WB (*n* = 3). (C) The expression of MEF2A was determined by IF (*n* = 3). (D) Cell viability was analyzed by CCK‐8 (*n* = 3). (E) The protein expression of p‐P65, P65, ZO‐1, Claudin‐1, and Occludin was determined by WB (*n* = 3). (F) The levels of inflammatory factors IL‐1β, TNF‐α, and IL‐6 were determined by ELISA (*n* = 3). (G) The protein expression of NLRP3, IL‐1β, and ASC was determined by WB (*n* = 3). Data are presented as mean ± SD ( ^∗^ *p* < 0.05,  ^∗∗^ *p* < 0.01,  ^∗∗∗^ *p* < 0.001).

**Figure:**
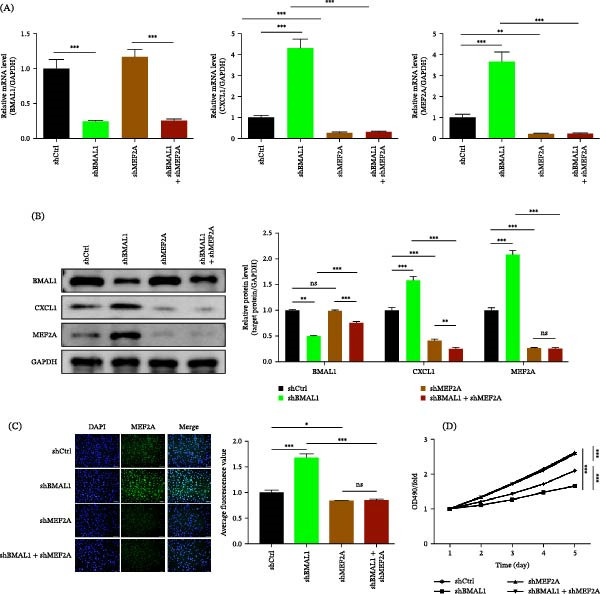

**Figure:**
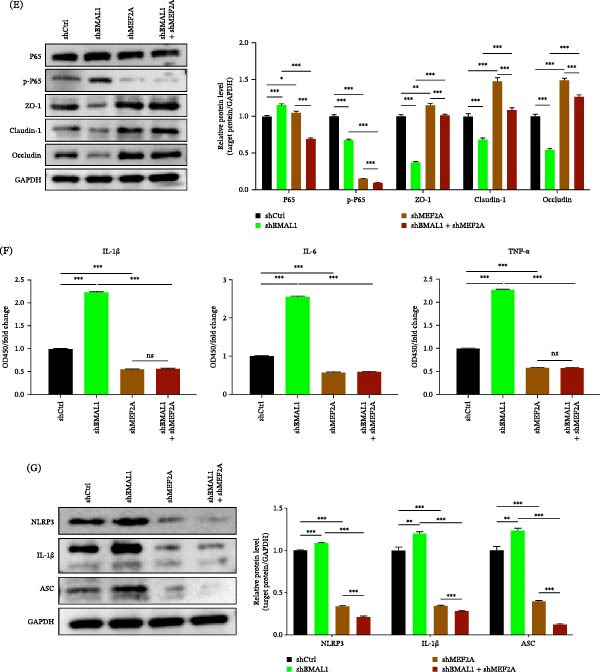

### 3.7. BMAL1 Ameliorated DSS‐Mediated UC Symptoms In Vivo

**Figure 7:**
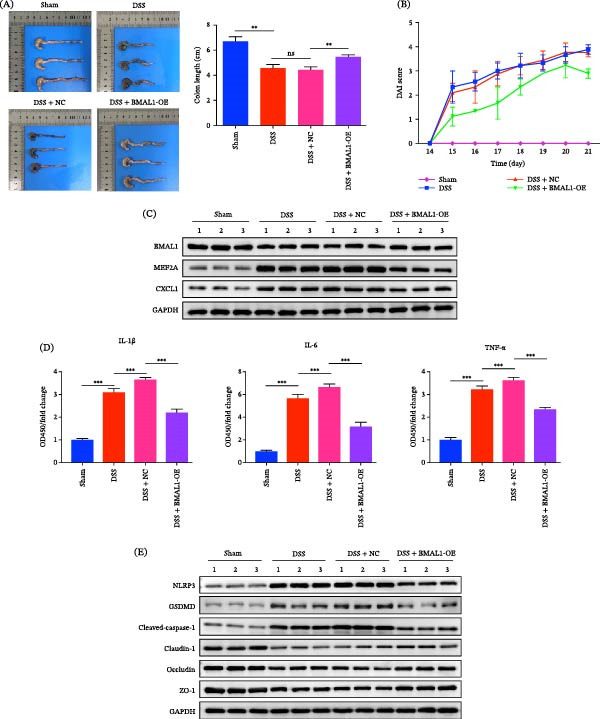
BMAL1 ameliorated DSS‐mediated UC symptoms in vivo. C57BL/6 mice (male, 6–8 weeks old) were equally divided into 4 groups, mice treated without DSS treatment(sham group, *n* = 5), mice injected with DSS (DSS group, *n* = 5), mice injected with DSS and NC lentivirus(DSS + NC group, *n* = 5), mice injected with DSS and BMAL1‐OE lentivirus (DSS + BMAL1‐OE group, *n* = 5). (A) Colon length of mice in each group was determined. (B) The disease activity index (DAI) score was examined on day 14–21. (C) The protein expression of BMAL1, CXCL1, and MEF2A in colon tissues was determined by WB. (D) The levels of inflammatory factors IL‐1β, TNF‐α, and IL‐6 in colon tissues were determined by ELISA. (E) The protein expression of NLRP3, Gasdermin D, cleaved caspase‐1, ZO‐1, Claudin‐1, and Occludin in colon tissues was determined by WB. Data are presented as mean ± SD. ( ^∗∗^ *p* < 0.01,  ^∗∗∗^ *p* < 0.001).

Finally, we established a DSS‐induced in vivo ulcerative colitis (UC) model to validate the functional role of BMAL1. In DSS‐treated UC mice, BMAL1 overexpression ameliorated DSS‐induced colon shortening (Figure 7A) and partially reversed the elevated disease activity index (DAI) scores (Figure 7B). DSS treatment significantly reduced BMAL1 expression while increasing CXCL1 and MEF2A levels in colon tissues; however, BMAL1 overexpression reversed these changes (Figure 7C). Compared to sham controls, DSS markedly enhanced the secretion of inflammatory cytokines (IL‐1β, TNF‐α, and IL‐6), which was attenuated by BMAL1 overexpression (Figure 7D). Furthermore, BMAL1 overexpression reversed the DSS‐mediated upregulation of pyroptosis markers (NLRP3, Gasdermin D, and cleaved caspase‐1) and the downregulation of TJ proteins (ZO‐1, Claudin‐1, and Occludin) in colon tissues (Figure 7E). These results demonstrate that BMAL1 overexpression alleviates UC symptoms by reducing colonic pyroptosis and restoring intestinal epithelial barrier integrity in vivo.

## 4. Discussion

**Figure 8:**
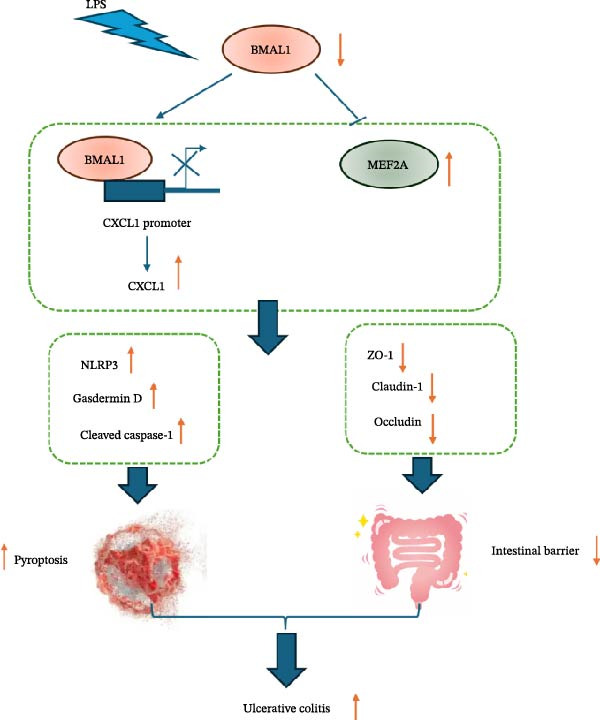
Schematic diagram of the molecular mechanism underlying the protective effect of BMAL1 on the colonic barrier. Overexpression of BMAL1 enhanced LPS‐induced intestinal epithelial barrier function and suppressed pyroptosis in intestinal epithelial cells. Mechanistically, on one hand, BMAL1 negatively regulated CXCL1 activity by inhibiting its promoter activity, thereby improving intestinal epithelial barrier function. On the other hand, BMAL1 significantly reduced the expression of MEF2A, which also contributed to the enhancement of intestinal epithelial barrier function.

In this study, we innovatively investigated the role and molecular mechanism of the circadian rhythm gene BMAL1 in intestinal epithelial barrier injury. The results indicated that BMAL1 expression levels were significantly reduced in UC tissues and in LPS‐induced MODE‐K cells. Overexpression of BMAL1 enhanced LPS‐induced intestinal epithelial barrier function and inhibited pyroptosis of IECs. Mechanistically, BMAL1 improved intestinal epithelial barrier function by inhibiting CXCL1 promoter activity, which direct negatively regulated CXCL1 expression. Proteomic studies revealed that BMAL1 significantly reduced the level of MEF2A. The protective effect of BMAL1 on intestinal epithelial barrier function was also mediated through the expression of MEF2A. Therefore, CXCL1 and MEF2A are parallel downstream pathways of BMAL1, and BMAL1 exerts its effects through these two parallel pathways. Hence, BMAL1 may serve as a potential target gene, providing a theoretical basis for the treatment of UC. The detailed mechanism of BMAL1 is illustrated in Figure 8.

Most notably, immune cell reactivity exhibits strong circadian rhythms [38]. Mechanistically, this may serve to maximize immune defenses during periods of heightened pathogen exposure and to promote tissue repair during rest periods [39, 40]. Most immune cell lineages possess intrinsic clocks, and in some instances, circadian mechanisms contribute to the development and differentiation of these lineages [41]. Therefore, maintaining circadian rhythmicity may aid in the treatment of immune system disorders. The pathogenesis of ulcerative colitis (UC), categorized as a chronic inflammatory bowel disease (IBD), has also been linked to disrupted immune responses. Consequently, circadian disorders may play a role in the progression of UC. For example, Jochum et al. [42] found that disruption of the intestinal circadian rhythm in colonic epithelial cells resulted in increased colitis and increased inflammatory mediators. Accordingly, in the present study, we also analyzed the expression of circadian rhythm‐related genes in tissues from UC patients. The results revealed that the mRNA and protein expression levels of the core biological clock genes CLOCK, BMAL1, CRY1, CRY2, PER1, and PER2 were significantly downregulated in the colon tissues of UC patients. This suggested that UC patients presented with circadian rhythm system disturbances in the intestinal, similar to previous studies. Combined with the results from the literature, the deletion of BMAL1 is closely related to the pathogenesis of colitis [24], so we focused on the role and mechanism of BMAL1 in UC. Not surprisingly, BMAL1 was also found to be lowly expressed in DSS‐induced mice and LPS‐induced MODE‐K cells. The above suggested that BMAL1 deficiency might aggravate UC progression. Recent studies have demonstrated that stimulation of the hypothalamic paraventricular nucleus alleviates intestinal damage in rats with ulcerative colitis [43], highlighting the gut–brain axis as a critical biological pathway in intestinal injury. Notably, the central circadian pacemaker located in the hypothalamic suprachiasmatic nucleus regulates peripheral circadian rhythms [44]. As a core circadian clock gene, BMAL1 undergoes central regulation in the brain and may ameliorate intestinal inflammation and barrier dysfunction through modulation of the brain–gut axis.

It is well known that intestinal epithelial barrier damage is closely associated with the progression of UC [45]. At the same time, circadian rhythm genes can modulate the integrity of the intestinal barrier. For example, previous studies have shown that in mice, knockout of circadian genes increases susceptibility to severe intestinal inflammation and epithelial dysregulation, thereby disrupting the intestinal barrier [20]. Intestinal barrier damage can be prevented by reversing circadian rhythm gene disruption [46]. However, the existing literature lacks studies on the specific mechanisms by which circadian rhythms are regulated at the intestinal epithelial barrier. Therefore, in the current study, we investigated whether the circadian rhythm gene BMAL1 improves intestinal epithelial barrier integrity by regulating the expression of TJ proteins (ZO‐1, Occludin, and Claudin1). In this study, we found that overexpression of BMAL1 restored the expression of ZO‐1, Occludin, and Claudin1 which was reduced by LPS, suggesting that BMAL1 could ameliorate intestinal epithelial barrier injury. In addition, the massive death of IECs can induce injury to the intestinal epithelial barrier. Notably, abnormal death of a significant number of IECs has been observed in a preclinical model of UC patients [12]. Pyroptosis, also known as inflammatory cell necrosis, is a novel form of programmed inflammatory cell death [47]. GSDME‐mediated pyroptosis of IECs has been reported to be prominent in UC tissues from both human and mouse models [48]. On one hand, the release of inflammatory cytokines such as IL‐1β and IL‐18 during the pyroptosis process can affect the expression of TJ proteins, thereby altering the permeability of the intestinal epithelium [49, 50]. On the other hand, the substantial death of IECs induced by pyroptosis also disrupts the intestinal epithelial barrier [51]. Therefore, alleviating pyroptosis in IECs could improve intestinal epithelial barrier dysfunction in UC. Consistent with previous reports, the present study found that LPS induced pyroptosis of IECs and that overexpression of BMAL1 reduced the pyroptosis index and the release of inflammatory factors. This suggested that BMAL1 might alleviate IECs pyroptosis to improve the function of the epithelial barrier.

CXC chemokines have pro‐inflammatory properties [52]. Previous studies have demonstrated that the CXC chemokine CXCL1 was a diagnostic biomarker of UC activity and high expression of CXCL1 exacerbated UC symptoms [32, 33]. Similar to previously reported results, the present study also found a high degree of CXCL1 expression in UC patient tissues and DSS‐induced mice. This suggested that high expression of CXCL1 was involved in the UC process. Moreover, we also found that the knockdown of CXCL1 protected IECs from LPS‐induced pyroptosis and alleviated intestinal epithelial functional impairment. Interestingly, BMAL1 negatively regulates CXCL1 by inhibiting the CXCL1 promoter activity, and we found that the protective effect of BMAL1 in IECs was achieved by regulating CXCL1 expression.

To investigate the molecular mechanisms regulated by BMAL1, this study utilized proteomics to identify key proteins influenced by BMAL1. Among these, MEF2A emerged as a significant gene, with BMAL1 knockdown leading to a marked increase in MEF2A expression. Elevated levels of MEF2A have been implicated in inflammatory regulatory processes [53]; for instance, its overexpression promoted the release of inflammatory factors during sepsis [54]. In addition, MEF2A interacts with STAT3 to promote inflammatory responses [55]. The present study found that knockdown of BMAL1 increased the expression of MEF2A. Furthermore, BMAL1 knockdown further exacerbated LPS‐induced pyroptosis and inflammatory cascades in MODE‐K cells. We hypothesize that BMAL1 may protect against LPS‐induced intestinal barrier dysfunction by negatively regulating MEF2A‐mediated inflammatory pathways, such as the STAT3 signaling axis. In summary, these findings suggest that BMAL1 protects MODE‐K cells through the negative regulation of MEF2A expression.

However, limitations remain in this study. On the one hand, other molecular mechanisms by which BMAL1 regulates the death of IECs need to be explored, such as ferroptosis, mitochondrial damage, and copper death. On the other hand, we acknowledge that the relatively small sample size (15 patients with ulcerative colitis and 15 controls) limits the statistical power of this study. Therefore, these findings should be considered exploratory and hypothesis‐generating and require validation in larger, well‐characterized prospective cohorts. Future studies should aim to correlate the expression of BMAL1 and CXCL1 with detailed clinicopathological parameters—including disease activity indices and treatment response—in larger patient cohorts to fully determine their translational significance. Furthermore, the regulatory effect of BMAL1 on MEF2A remains to be elucidated in future studies. The interaction between these two factors may be indirect or mediated through other mechanisms, warranting additional experimental validation. Nevertheless, it is undeniable that our study has improved our understanding of BMAL1 function and provided a theoretical basis for the treatment of UC.

Finally, to summarize, this study investigated the role and mechanism of BMAL1 in intestinal epithelial inflammation. The findings suggested that BMAL1 enhanced intestinal epithelial barrier function by direct negative regulation of CXCL1 or indirect negative regulation of MEF2A expression, as well as decreasing cell pyroptosis during intestinal epithelial inflammation. Therefore, BMAL1 may serve as a potential target gene, providing a theoretical basis for the treatment of UC.

## Author Contributions

**Xin Zhou:** analysis, interpretation, obtaining funding. **Jie Ji:** writing the article. **Wenhua Li:** data collection. **Wenxia Wu:** data collection. **Huanyan Zhang:** data collection. **Pingyu Gao:** critical revision of the article. **Wenjiao Ren:** writing the article. **Guanzhao Zong:** critical revision of the article. **Weiliang Jiang:** critical revision of the article. **Rong Wan:** data collection. **Kai Zhao:** statistical analysis. **Yongbin Ma:** conception and design. **Zhanjun Lu:** conception and design.

## Funding

This work was supported by the Science and Technology Plan (Apply Basic Research) of Changzhou Science and Technology Bureau (Grant CJ20230003); the Natural Science Foundation of Jiading District Science and Technology Commission (Grant JDKW‐2023‐0019); and the Science and Technology Project of Jiading Hospital, Shanghai General Hospital (Grant 202137A).

## Disclosure

The final manuscript has been reviewed and approved by all the authors.

## Ethics Statement

The study was approved by the Ethics Committee of Jintan Hospital (Approval Number 2023005). The procedures used in this study adhere to the tenets of the Declaration of Helsinki. All procedures of animal experiments were approved by the Shanghai General Hospital Clinical Center Laboratory Animal Welfare and Ethics Committee (Approval Number 2025AWS096). The animal experiments in this study followed the ARRIVE reporting guidelines.

## Consent

All participants in this study have informed consent. The study is reported in accordance with ARRIVE guidelines.

## Conflicts of Interest

The authors declare no conflicts of interest.

## Supporting Information

Additional supporting information can be found online in the Supporting Information section.

## Supporting information


**Supporting Information 1** Table S1: Patient clinical information.

**Supporting Information 2** Figure S1: Fluorescent images of cells after lentivirus transfection. MODE‐K cells were transfected with NC‐OE, BMAL1 ‐OE, shCtrl, shBMAL1, shCXCL1, and shMEF2A lentiviruses, and images of the cells were taken under light microscope and fluorescence microscope to demonstrate that the cells were stably transfected.

**Supporting Information 3** Figure S2: Screening of BMAL1‐related proteins based on proteome sequencing. MODE‐K cells were transfected with shCtrl or shBMAL1 lentivirus and subsequently analyzed by proteomics. (A) Volcano plot of differentially expressed proteins. (B) Bubble diagram of GO analysis of differentially expressed proteins. (C) Bubble diagram of KEGG analysis of differentially expressed proteins. (D) The mRNA expression of HDAC8, STAT5B, MEF2A, and ZNF281 was determined by RT‐qPCR. Data are presented as mean ± SD.  ^∗^ *p*  < 0.05,  ^∗∗^ *p*  < 0.01,  ^∗∗∗^ *p*  < 0.001.

**Supporting Information 4** Table S2: Based on proteomic analysis, all differentially expressed proteins in the BMAL1 knockdown group were screened.

## Data Availability

The datasets used and/ or analyzed during the current study are available from the corresponding author upon reasonable request.
