# Supplementary figures and images for: Deletion of Circadian Rhythms Gene BMAL1 Impairs the Intestinal Epithelial Barrier and Exacerbates Intestinal Inflammation by Inducing Pyroptosis
Source: Mediators Inflamm. 2026 Jul 22;2026:9930240. doi: 10.1155/mi/9930240 (PMC13389442; doi:10.1155/mi/9930240)

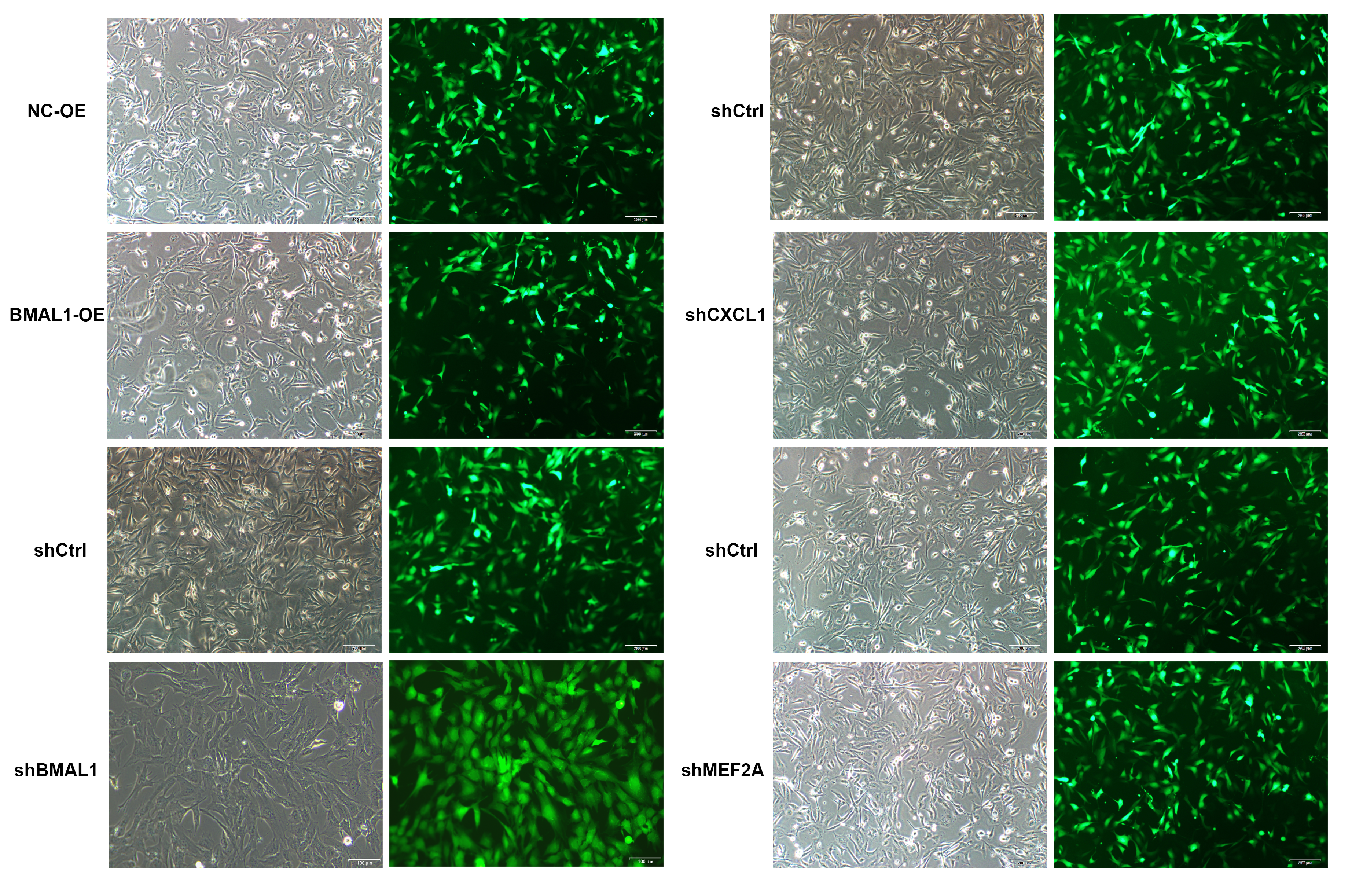

Supplement: Supplementary file 2 — Supporting Information 2 Figure S1: Fluorescent images of cells after lentivirus transfection. MODE‐K cells were transfected with NC‐OE, BMAL1 ‐OE, shCtrl, shBMAL1, shCXCL1, and shMEF2A lentiviruses, and images of the cells were taken under light microscope and fluorescence microscope to demonstrate that the cells were stably transfected. [file MI-2026-9930240-s002.tif]

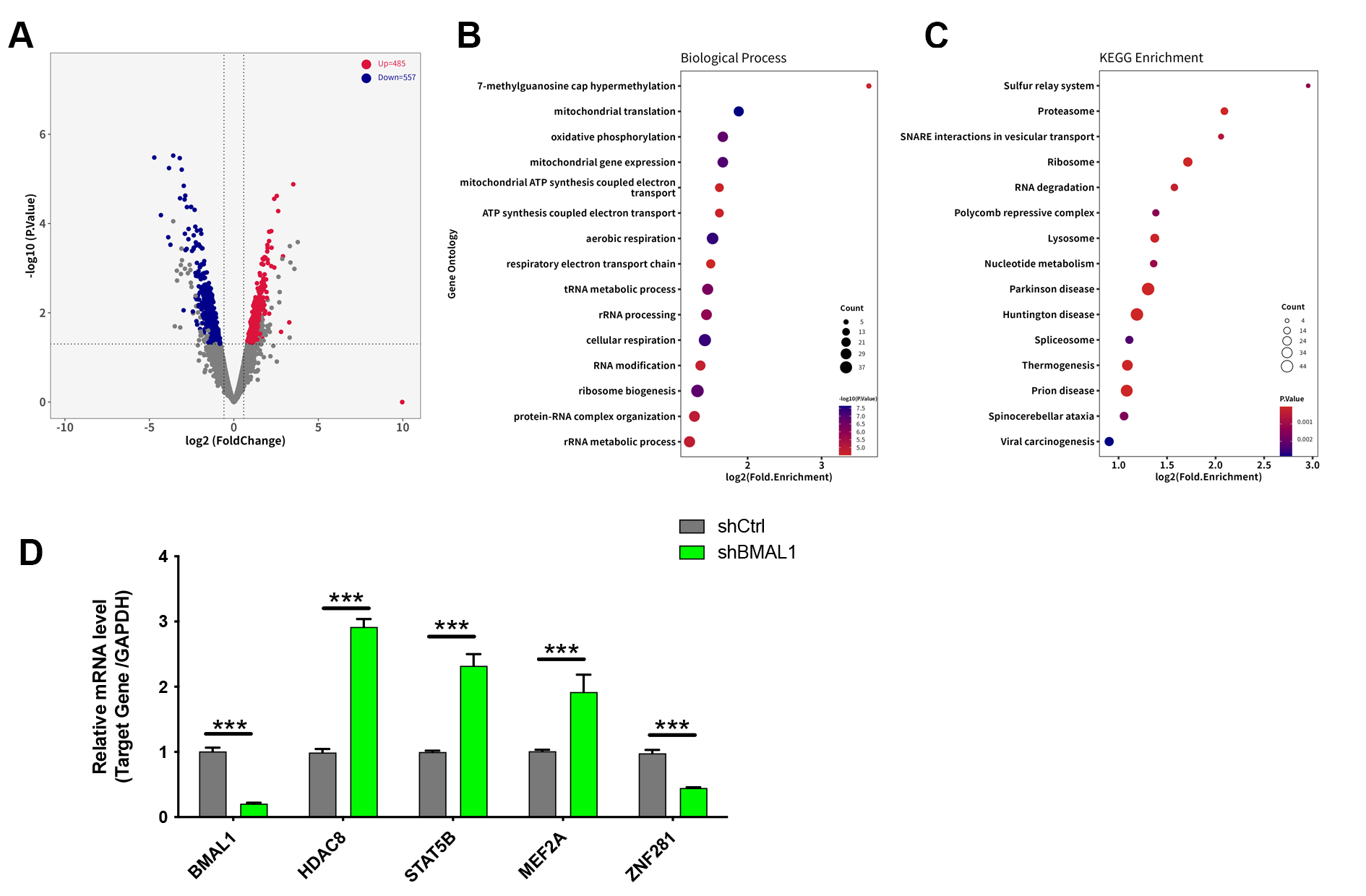

Supplement: Supplementary file 3 — Supporting Information 3 Figure S2: Screening of BMAL1‐related proteins based on proteome sequencing. MODE‐K cells were transfected with shCtrl or shBMAL1 lentivirus and subsequently analyzed by proteomics. (A) Volcano plot of differentially expressed proteins. (B) Bubble diagram of GO analysis of differentially expressed proteins. (C) Bubble diagram of KEGG analysis of differentially expressed proteins. (D) The mRNA expression of HDAC8, STAT5B, MEF2A, and ZNF281 was determined by RT‐qPCR. Data are presented as mean ± SD. ∗ p < 0.05, ∗∗ p < 0.01, ∗∗∗ p < 0.001. [file MI-2026-9930240-s003.tif]
